# Poly(I:C)/Alum Mixed Adjuvant Priming Enhances HBV Subunit Vaccine-Induced Immunity in Mice When Combined with Recombinant Adenoviral-Based HBV Vaccine Boosting

**DOI:** 10.1371/journal.pone.0054126

**Published:** 2013-01-15

**Authors:** Xia Chuai, Hong Chen, Wen Wang, Yao Deng, Bo Wen, Li Ruan, Wenjie Tan

**Affiliations:** 1 National Institute for Viral Disease Control and Prevention, Chinese Center for Disease Control and Prevention, Beijing, People’s Republic of China; 2 Department of Microbiology, Hebei Medical University, Shijiazhuang, Heibei Province, People’s Republic of China; 3 College of Life Science, Jilin University, Changchun,Jinlin Province, People’s Republic of China; University of Illinois at Chicago, United States of America

## Abstract

**Background:**

Virus-specific cellular immune responses play a critical role in virus clearance during acute or chronic HBV infection. Currently, the commercially available HBV vaccine is combined with alum adjuvant, which stimulates mainly Th2 immune responses. Therefore, development of new therapeutic HBV vaccine adjuvants and immune strategies that also promote Th1 and CTL responses is urgently needed.

**Methodology/Principal findings:**

To improve the immunity induced by the novel HBSS1 HBV vaccine, we evaluated the ability of adjuvants, including alum, CpG and polyriboinosinic polyribocytidylic acid [poly(I:C)], to enhance the response when boosted with the recombinant adenoviral vector vaccine rAdSS1. The immune responses to different adjuvant combinations were assessed in C57BL/6 mice by enzyme-linked immunosorbent assay (ELISA), ELISpot and cytokine release assays. Among the combinations tested, a HBV protein particle vaccine with CpG/alum and poly(I:C)/alum priming combinations accelerated specific seroconversion and produced high antibody (anti-PreS1, anti-S antibody) titres with a Th1 bias. After boosting with recombinant adenoviral vector vaccine rAdSS1, both groups produced a strong multi-antigen (S and PreS1)-specific cellular immune response. HBSS1 immunisation with poly(I:C)/alum priming also generated high-level CD4^+^ and CD8^+^ T cell responses in terms of Th1 cytokines (IFN-γand IL-2).

**Conclusions:**

The protein-vaccine HBSS1 with mixed poly(I:C)/alum adjuvant priming, followed by a rAdSS1 vaccine boost, maximises specific antibody and Th1-biased cellular immune responses. This regime might prove useful in the development of HBV therapeutic vaccines. Furthermore, this promising strategy might be applied to vaccines against other persistent infections, such as human immunodeficiency virus and tuberculosis.

## Introduction

Hepatitis B virus (HBV) infection is a public health problem. Over 350 million people globally are chronically infected with HBV, and about 25% of those die from chronic active hepatitis, cirrhosis, or hepatocellular carcinoma [1]–[3]. Interferon-α and nucleoside analogues are the two main types of antiviral medicines used to treat chronic HBV. Interferon has a direct antiviral effect, but with only 20–40% efficacy. Although nucleoside analogues suppress HBV replication and transcription, they are not HBV-specific, and often cause side effects or result in decreased efficacy due to drug resistance [4]. The currently available recombinant subunit HBV vaccines are safe and efficacious for prevention; however, due to the lack of suitable adjuvants, they have no effect on the clearance of HBV among existing HBV carriers or patients. Therefore, there is a pressing need to develop a therapeutic vaccine to prevent, control or cure chronic HBV infection [5]. A successful HBV therapeutic vaccine would induce the activation of CD4+ T cells with Th1 bias to secrete anti-viral cytokines and promote CD8+ T cell activity. Consequently, CD8+ T cells would clear virus through both cytolytic (CTL) and non-cytolytic (anti-viral cytokines) activities [5], [6].

The currently used commercially available HBV vaccine is combined with alum adjuvant, which is recognised as a stimulator of Th2 immunity [7]; however, it does not stimulate robust Th1 immunity or enhance the CTL responses that are critical to virus clearance during acute or chronic HBV infection. Therefore, it does not meet current demands for use in a therapeutic vaccine [5], [6]. Substantial efforts have been devoted in recent years to developing a new generation of potent and safe adjuvants [6]. Recently, much work has focused on adjuvants that signal through pattern recognition receptors (PRRs), including Toll-like receptors (TLRs) [8]–[10]. Some TLR ligands or agonists such as CpG oligodeoxynucleotide (ODNs) (TLR9 ligands) [11]–[13] and poly(I:C) (TLR3 agonist) [14], [15] can stimulate the production of pro-inflammatory cytokines/chemokines and type I IFNs that increase the host’s ability to eliminate the pathogen [8], [9]. This innate immune response also supports the subsequent development of adaptive immunity, and thus can be harnessed to accelerate and enhance the induction of vaccine-specific responses [10]. CpG ODNs, which are short synthetic DNA sequences consisting of unmethylated CG dinucleotides, are currently being developed as vaccine adjuvants that function by mimicking the effects of bacterial DNA [11], [12], [13]. Synthetic CpG ODNs activate the immune system by signalling through TLR9 expressed on B cells and plasmacytoid dendritic cells in humans, which triggers both innate and adaptive immunity. As an adjuvant, CpG ODN promotes the Th1-type immune responses that play a key role in HBV clearance. Polyriboinosinic polyribocytidylic acid [poly(I:C)], a synthetic dsRNA that mimics the effects of naturally occurring dsRNA and the TLR3 agonist, is frequently used as an adjuvant in both antitumor treatment and vaccine development [14], [15]. A major mechanism underlying the strong adjuvant function of poly(I:C) in mice is that it is superior at inducing systemic type I IFN, which has many immune stimulatory roles for both T and B lymphocytes; furthermore, dendritic cells (DCs) have the potential to bridge the gap between innate and adaptive immunity [16]. Thus, these two adjuvants may be used to enhance HBV vaccine immune responses, and especially cell-mediated immune responses, against persistent HBV infection. To our knowledge, there are few reports of immunity associated with protein vaccines formulated with mixed TLR agonist [CpG or poly(I:C)] and alum adjuvants.

Our preliminary data suggested that the HBSS1 vaccine, which contained S (1–223 aa), PreS1 (21–47 aa) and a recombinant vaccinia virus (Tiantan) RVJSS1, elicits specific antibody and multi-antigen (PreS1, and S)-specific cellular immune responses, and generates more significant levels of both CD4^+^ and CD8^+^ T cell responses for Th1 cytokines (TNF-α and IFN-γ) [17]. Here, we tested the immunity effects when mixed adjuvants [alum and CpG; alum and Poly(I:C)] were used in combination with the HBSS1 protein vaccine. In addition, because of their strong immunogenicity, their ability to transduce antigen-presenting cells (APCs) and elicit strong B and T cell immune responses to target antigens, adenoviral vectors are attractive carriers for genetic vaccines [18]. We constructed a recombinant replication-defective adenovirus serotype 5 vector expressing the fusion protein SS1 (rAdSS1) and employed it in the ‘prime–boost’ regime evaluated in this study. Our data showed that HBSS1 in conjunction with mixed adjuvant priming and rAdSS1 boosting represents a novel promising avenue of HBV therapeutic vaccine development.

## Materials and Methods

### Generation of Vaccine Candidates

**Figure 1:**
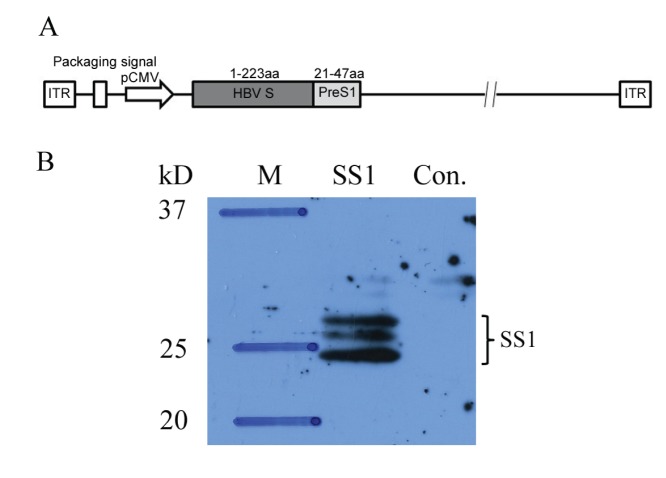
Characterisation of recombinant adenovirus rAdSS1. (**A**) Schematic representation of recombinant adenovirus viral vectors encoding HBV S and PreS1 fusion genes. ITR, inverted terminal repeat. (B) Western blot detection of SS1 fusion protein expression in HEK293 cells infected with rAdSS1 using specific rabbit anti-PreS1 polyclonal antibodies. The bands of the expressed SS1 proteins are indicated by arrowheads.

The HBV S (aa: 1−223) and PreS1 (aa: 21−47) fusion gene fragments [17] were used to construct the rAdSS1 (Figure 1). This adenovirus type 5 construct is a prototypic first-generation vector containing an E1 deletion replaced with the HBV SS1 expression cassette. This E1 deletion renders the vector replication-defective; however, when E1 is provided *in trans* in HEK293, the vector is efficiently propagated. The rAdSS1 was amplified in HEK293 cells and purified as report previously [19]. To confirm expression of the HBV fusion protein SS1, HEK293 cells were infected with rAdSS1, and cell lysates were subjected to Western blot at 48 h post-infection using specific rabbit anti-PreS1 polyclonal antibodies (Alpha Co., Shanghai,China). The HBsAg in culture supernatants of infected cells were determined by enzyme-linked immunosorbent assay (ELISA). The critical value (cut off) = 2.1 × N, in which N is the average OD of the negative control, and S is the sample OD. Samples were considered positive when S/N ≥2.1.

HBSS1 particle subunit vaccine containing S (1−223 aa) and PreS1 (21−47 aa) was expressed in Chinese hamster ovary (CHO) cells [20]. To determine whether the protein vaccine HBSS1 formed particles, we performed electron microscopy using negative staining as described previously [17]. The purity of HBSS1 was >95%, and the antigen content was 40 µg/mL.

### Adjuvants

The HBSS1 was formulated with different adjuvant(s) immediately prior to immunization as previous reports[11]–[16], [21]–[25], The HBSS1 single dose (100 µL) was 1.25 µg, and with or without 100 µg alum (named A), 10-µg CpG (named C) or 50 µg poly(I:C) (named P) or mixed adjuvant (A+C or A+P) (Table 1). Aluminium hydroxide (alum) adjuvant was kindly provided by the North China Pharmaceutical Group Corporation GeneTech Biotechnology Development Company. The ODN motif containing unmethylated cytosine preceding guanosine (CpG) (5′-TCCATGACGTTCCTGACGTT-3′) was synthesised with a full phosphorothioate backbone by TAKARA BIO INC. Poly(I:C) was purchased from Sigma (St. Louis, MO), and dissolved in dimethyl sulfoxide.

**Table 1:** Vaccination groups and administration strategy.

Group	Prime (0 and 4 weeks)		Boost (14 weeks)
	Immunogen	Adjuvant(s)	Immunogen
1	NS	–	NS
2	Adjs(A+C+P)	–	–
3	NS	–	rAdSS1
4	HBSS1	–	rAdSS1
5	HBSS1	A	rAdSS1
6	HBSS1	C	rAdSS1
7	HBSS1	P	rAdSS1
8	HBSS1	A+C	rAdSS1
9	HBSS1	A+P	rAdSS1

A, alum; C, CpG; P, poly(I:C).

### Animals and Immunisation

**Figure 2:**
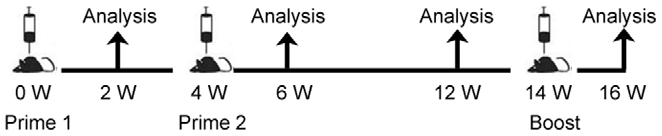
Immunisation of C57BL/6 mice. Each group (12 mice/group) was primed twice with HBSS1 together with various adjuvant combinations at weeks 0 and 4. At week 14, all immunised groups were boosted with rAdSS1. Humoral and cellular immune responses were evaluated at the indicated times.

Female C57BL/6 mice, 6–8 weeks of age (Animal Care Centre, Chinese Academy of Medical Science, Beijing, China), were randomly distributed into nine groups (Table 1), and primed twice with HBSS1 combined with different adjuvants at 4-week intervals. All immunised groups were boosted with rAdSS1 at 10-week intervals (Figure 2). Controls included mice immunised with saline(NS) or all the adjuvants(Adjs,A+C+P). The viral doses of rAdSS1 were 1 × 10^8^ vp/mouse. Immunisations were performed by intramuscular injection. A schematic of the vaccination and analysis timeline is shown in Figure 2. All experiments were conducted in accordance with the Institutional Animal Care and Use Committee (IACUC)-approved protocol.

### ELISA (Enzyme-linked Immunosorbent Assay)

Mouse sera were tested for an HBV-antigen-specific IgG antibody (anti-S and anti- PreS1) response using a commercially available ELISA Kit (Kehua, Shanghai, China) according to the manufacturer’s instructions and as described previously [17]. The optical density at 450 nm was read using an ELISA reader (Bio-Rad). The end titre was determined when the reading of the last serum dilution was 2.1-fold greater than that in the negative control wells that contained normal mouse sera.

Determination of IgG subclasses was conducted as described previously [17]. Serum samples from immunised mice were diluted 1∶100, and the IgG subclass was determined using a murine antibody isotyping ELISA kit (Sigma). Biotinylated rat anti-mouse IgG1 (1∶1000) or biotinylated rat anti-mouse IgG2a and IgG2b (1∶2000) were used. The development of colour occurred at room temperature and was read at 450 nm. The cut-off value was set at 2.1-fold that of the negative control.

### ELISpot Analysis

To evaluate the antigen-specific T cell responses induced by our immunisation regimen, an IFN-γ ELISpot assay was performed according to the manufacturer’s instructions and as described previously [17]. Briefly, multiscreen 96-well plates were coated overnight at 4°C with 100 µL per well of 5 µg/mL anti-mouse gamma interferon antibody (IFN-γ) (BD Pharmingen) in PBS and blocked for 2 h at room temperature. Freshly isolated mouse splenocytes (5 × 10^5 ^per well) were added to wells in triplicate with4µg/mL peptides separately, as described previously [17]. Next, a biotinylated detection antibody (BD Pharmingen) and streptavidin–horseradish peroxidase were added, and blots were developed by addition of AEC (3-amino-9-ethylcarbazole) substrate solution to yield a coloured spot after a 20–40-min incubation at RT in the dark. Finally, IFN-γ spot-forming cells (SFCs) were counted. The results are expressed as the number of SFCs per 10^6^ input cells. The number of peptide-specific IFN-γ-secreting T cells was calculated by subtracting the background (no-peptide) control value from the established SFC count.

### Cytokine Release Assay

Mouse splenocytes were isolated as above, plated at a density of 1 × 10^6^ per plate and incubated at 37°C in the presence of 5% CO_2_, with or without 4µg/mL of the HBV PreS1 and S antigen-relevant peptides used above. After 48-h incubation, culture supernatants were harvested and IFN-γ, IL-2, IL-4 and TNF-α levels were determined using commercial mouse immunoassay ELISA kits (Neobioscience), according to the manufacturer’s instructions. The concentrations of cytokines detected in the samples were determined from standard curves.

### Statistical Analysis

Statistical analyses were conducted using the one-way ANOVA analysis function in the SPSS software package SSPS, ver. 17.0. Values of *p*<0.05 were considered to indicate statistical significance.

### Ethical Approval

This study was carried out in strict accordance with the recommendations in the Guide for the Care and Use of Laboratory Animals according to the regulation in the People's Republic of China. All animal protocols (#2011036)were approved by the Institutional Animal Care and Use Committee (IACUC) of China CDC. All procedures were performed under isoflurane anesthesia, and all efforts were made to minimize suffering.

## Results

### Construction, Expression, and Identification of the Recombinant Adenoviral 5 (rAd5)- Based SS1 Vaccine, rAdSS1

We constructed an rAd5-based vaccine (rAdSS1) expressing the HBV fusion gene SS1 (Figure 1A). Expression of the target proteins was confirmed by Western blot using a rabbit anti-PreS1 polyclonal antibodies, which recognize HBSS1 protein with 3 bands of different molecular weights, two of bands were showed as previous report size(∼24 kD, ∼27 kD),the third band(∼26 kD) might represent another glycoylated form of HBSS1 protein produced in this system(Figure 1B). The culture supernatants of rAdSS1-infected cells were HBsAg-positive (data not shown).

### Induction of a Robust Humoral Immune Response by Adjuvant-primed HBSS1

**Figure 3:**
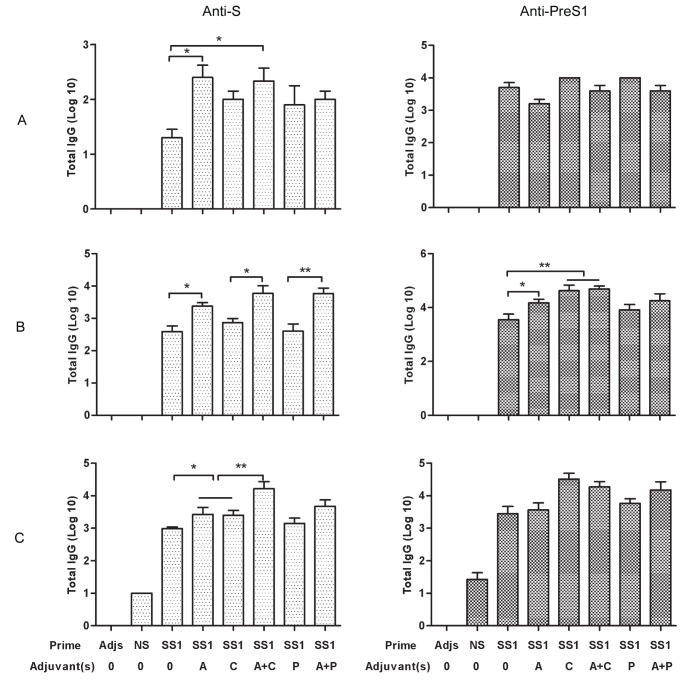
Antibody responses in immunised mice. (**A**) Antisera were collected at 2 weeks after the 2^nd^ priming; (**B**) Antisera were collected at 2 weeks before rAdSS1 boosting; (**C**) Antisera were collected at 2 weeks after rAdSS1 boosting. Total IgG titres specific for the HBV antigen were determined by ELISA as described in the Methods section. Statistical significance was analysed and shown as **p<0.01 and *p<0.05. SS1: HBSS1. A, alum; C, CpG; P, poly(I:C). Adjs: control group with adjuvants mixture.

Anti-S/PreS1-specific humoral immune responses in mice immunised with HBSS1 alone or with various adjuvants were subjected to enzyme-linked immunosorbent assay (ELISA). Serum samples collected at weeks 2, 6, 12 and 16 were tested in duplicate (Figure 2). Two weeks after the first HBSS1 priming, higher anti-PreS1 and anti-S seroconversion was induced in mice immunised with HBSS1 with CpG or poly(I:C) alone and with mixed adjuvant [alum together with CpG or poly(I:C)], compared to that induced in mice immunised with HBSS1 alone (Table 2). All immunised mice developed detectable anti-S/PreS1 antibodies after the second priming (Figure 3). Specific antibody titres were also determined at two and ten weeks after the second HBSS1 boosting and two weeks after rAdSS1 boosting. Two weeks after the second boosting, total anti-S/PreS1 antibody levels in all immunised groups were enhanced significantly. Additionally, slightly increased titres of IgG antibody were detected at week 10 after the second HBSS1 boosting, especially in groups immunised with mixed adjuvant (A+C, or A+P), compared with that at week 2 after the second HBSS1 boosting. The anti-S and anti-PreS1 antibodies reached higher titres (1∶1000–1∶10000) that were maintained for a long period in groups with mixed adjuvant (A+C, or A+P), compared with groups primed twice with different individual adjuvant(A, C, or P). After rAdSS1 boosting, all groups maintained higher antibody levels, but with no significant increase compared with that at 10 weeks after the second HBSS1 boosting.

**Table 2:** Total antibody-positivity rates after the 1^st^ and 2^nd^ HBSS1 priming.

Group	Immunogen	Adjuvant(s)	Anti-S		Anti-preS1	
			Prime 1	Prime 2	Prime 1	Prime 2
1	NS	–	0	0	0	0
2	Adjs(A+C+P)	–	0	0	0	0
3	NS	–	0	0	0	0
4	HBSS1	–	1/12	12/12	8/12	12/12
5	HBSS1	A	1/12	12/12	9/12	12/12
6	HBSS1	C	11/12	12/12	12/12	12/12
7	HBSS1	P	9/12	12/12	12/12	12/12
8	HBSS1	A+C	8/12	12/12	12/12	12/12
9	HBSS1	A+P	9/12	12/12	12/12	12/12

A, alum; C, CpG; P, poly(I:C).

These data suggest that two immunisation doses of HBSS1 protein vaccine with alum together with CpG or poly(I:C) adjuvants accelerated seroconversion and enhanced the antibody response. Both anti-S and anti-PreS1 antibodies were present at higher levels that were maintained for a longer period, and rAdSS1 boosting did not markedly enhance the antibody response.

### Induction of IgG Subtype Switching by HBSS1 Plus Priming with CpG or poly(I:C)

**Figure 4:**
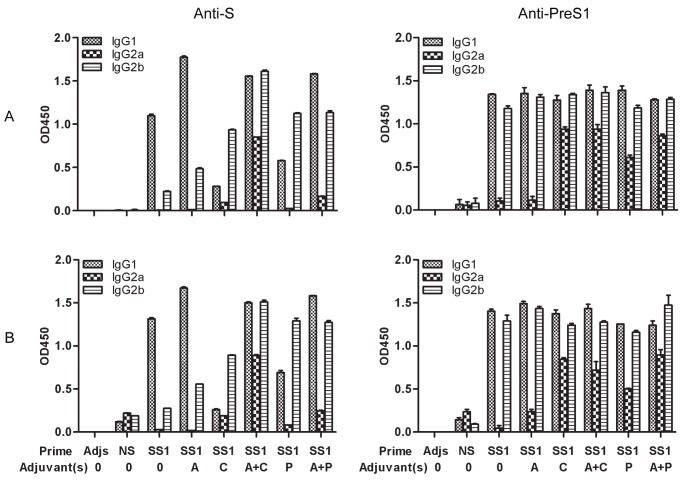
Subtype analysis of the HBV antigen-specific IgGs (anti-S and anti-PreS1) in sera of immunized mice before and after rAdSS1 boosting. (**A**) Sera were collected at 2 weeks after the 2^nd^ priming; (**B**) Sera were collected at 2 weeks after rAdSS1 boosting. Sera were diluted 1∶100. Bars indicate the average OD at 450 nm (OD_450_) of each group. SS1: HBSS1. A, alum; C, CpG; P, poly(I:C). Adjs: control group with adjuvants mixture.

The IgG subtype induced in each group was evaluated by ELISA. Serum samples were collected two weeks before and after rAdSS1 boosting and diluted 1∶100. As shown in Figure 4, IgG1 subtypes of antibodies against S were detected primarily in mice immunised with HBSS1 alone or with alum adjuvants, before and after rAdSSI boosting. In contrast, IgG2b or IgG2b plus IgG2a were detected primarily in the HBSS1 with CpG or poly(I:C) adjuvants groups, before and after rAdSS1 boosting. Additionally, the antibodies against PreS1 in mice immunised with HBSS1 alone or with alum adjuvant were principally IgG1 and IgG2b, whereas the anti-PreS1 IgG2a titre was increased in groups immunised with HBSS1 with CpG or poly(I:C) alone and alum together with a CpG or poly(I:C) combination. In summary, alum together with CpG or poly(I:C) adjuvants induced a balanced Th1- and Th2-type immune response, and rAdSS1 boosting enhanced the Th1-type immune response.

### The Antigen-specific Cellular Immune Response was Maximised by Priming with HBSS1 with Alum and CpG or Alum with poly(I:C) Followed by rAdSS1 Boosting

**Figure 5:**
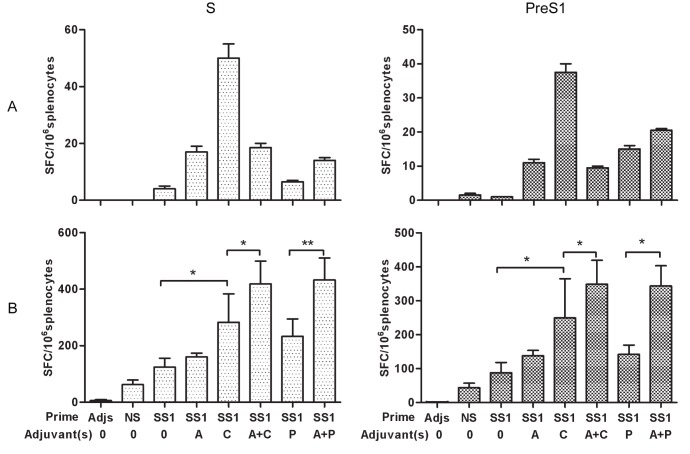
ELISpot analysis of IFN-γ secretion in mouse splenocytes. (A ) Splenocytes were isolated at weeks 6 after the 2nd priming; (B) Splenocytes were isolated at weeks 16 after the rAdSS1 boost. Data are expressed as spot-forming cell (SFC) responses to S and PreS1 peptide pools, and are presented as means with SEM. Significant *p* values between vaccinated groups are shown as * *p*<0.05, and ** *p*<0.01. SS1: HBSS1 A, alum; C, CpG; P, poly(I:C). Adjs: control group with adjuvants mixture.Data are shown as means ± SE or ± SEMs.

To assess the cellular immune response (CMI) elicited by our regimens, we next determined the frequencies of IFN-γ-producing cells at the single-cell level by ELISpot assay. Mouse spleen lymphocytes were stimulated with S or PreS1 peptide arrays as described previously. As shown in Figure 5A, two weeks after the second HBSS1 priming, both the S- and PreS1-specific SFC readings of all groups were low. Compared with the two-dose vaccination, the frequency of IFN-γ-producing cells in spleens from rAdSS1-boosted mice was dramatically increased (Fig. 5B). The numbers of IFN-γ secreting cells generated in response to S and PreS1 in groups primed with HBSS1 with CpG or with mixed adjuvants were higher than that in groups primed with HBSS1 alone or with alum adjuvants (*p*<0.01 or *p*<0.05). Therefore, rAdSS1 boosting enhanced the HBSS1-primed antigen-specific cellular immune response. Furthermore, the antigen-specific cellular immune response was maximised by HBSS1 priming with mixed adjuvant (alum and CpG or alum with poly(I:C)) and rAdSS1 boosting.

### IFN-γ and IL-2 Secretion in the HBSS1 with Alum and Poly(I:C)-primed Group

**Figure 6:**
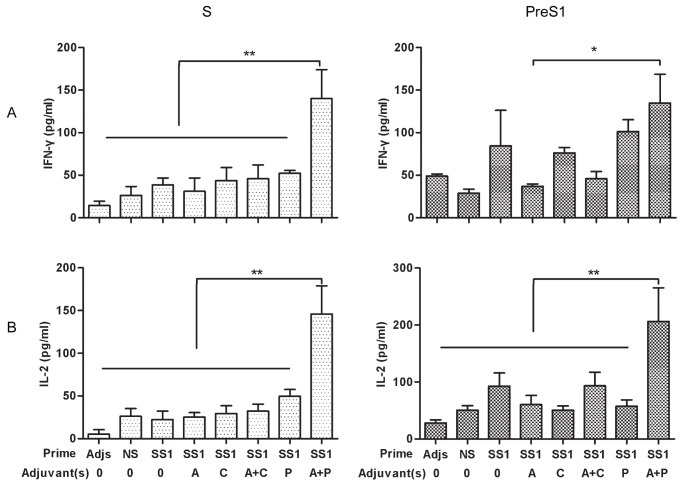
In vitro cytokine production by splenocytes from immunized mice after rAdSS1 boosting. (A) IFN-γ; (B) IL-2. Supernatants were collected at 48h after HBV S and PreS1 peptide pool stimulation and cytokines were quantified by ELISA. IFN-γand IL-2 were detected and data were shown as means±SEMs. Statistical differences between groups were determined, and differences are shown as *p<0.05 and **p<0.01. SS1: HBSS1 A, alum; C, CpG; P, poly(I:C). Adjs: control group with adjuvants mixture.

To determine whether T helper cells were activated, we measured cytokines (IL-2, IFN-γ, IL-4, and TNF-α) in the culture supernatants of mouse splenocytes after antigen-relevant peptides incubation. Compared with control cultures, no significant production of IL-4 and TNF-α was identified in any group (data not shown). As shown in Figure 6, IFN-γ and IL-2 levels in the HBSS1 with alum and poly(I:C) group were significantly higher than those in the other groups stimulated with S peptide after rAdSS1 boosting (*p*<0.01). Additionally, the IL-2 level was notably greater in the group immunised with PreS1 peptide in alum and poly(I:C) than that in the other groups (*p<*0.01). The IFN-γ level was higher in the group immunised with PreS1 peptide with alum and Poly(I:C), compared to that in the HBSS1 alone, alum or poly(I:C) groups (*p*<0.05). Therefore, immunisation with HBSS1 with alum and poly(I:C) promoted IFN-γ and IL-2 production, resulting in an enhanced antigen-specific T cellular immune response.

## Discussion

To compensate for the poor immunogenicity of subunit antigens, adjuvants have often been used to supplement immunogens. CpG and poly(I:C) are both toll-like receptor (TLR)-based adjuvants and link innate and adaptive immunity by signalling through a combination of PRRs [8]–[10], [21]. Here, novel recombinant HBSS1 particle antigen was formulated with the adjuvants, alum, CpG and poly(I:C), and also with combinations of alum and CpG, or of alum and poly(I:C). The antigen-adjuvant formulation was administered to mice as a prime–boost regimen. We investigated HBSS1 priming with different adjuvants in terms of their ability to elicit antibody and T-cell immune responses. To our knowledge, our data are the first to reveal that the HBV subunit vaccine in combination with alum and poly(I:C) induces an unusually potent immunity in mice boosted with a recombinant adenoviral-based HBV vaccine.

We previously constructed a novel HBV protein-particle vaccine, HBSS1, which was derived from the CHO system [20]. Compared with vaccines that contain only the S region, this novel HBSS1 vaccine induced both S- and PreS1-specific humoral and cellular immune responses in mice [17], [20]. The protein particle HBSS1 vaccine with CpG/alum or poly(I:C) did not produce higher antigen-specific cellular immune responses; therefore, we constructed rAdSS1 to boost after two protein vaccine immunisations. The adenovirus-based vaccine elicited vigorous and sustained humoral and T cell responses against the incorporated antigen, which is considered to be important for the clearance of persistent viral diseases [18], [19]. Our data indicated that rAdSS1 boosting enhanced the S and PreS1 antigen-specific SFC readings in each HBSS1-primed group with or without adjuvant (s), but the SFC readings varied in each group. The differences in each group were likely due to the adjuvants used in the priming immunisation.

Alum is included as an adjuvant in the currently used commercial HBV vaccines. Alum is recognised as a stimulator of Th2 immunity but does not induce cytotoxic T lymphocyte (CTL) responses to protect from viral infection [7]; thus, the development of new adjuvants that enhance both cell- and antibody-mediated immunity is crucial for control of chronic HBV infection. CpG and poly(I:C) are effective adjuvants that link innate and adaptive immunity by signalling through a combination of PRRs [8]–[10], [21]. Triggering via the TLR stimulates the production of pro-inflammatory cytokines/chemokines and type I IFNs that increase the host’s ability to eliminate the pathogen [8]–[10], [21]. This innate immune response also supports the subsequent development of adaptive immunity, and thus can be harnessed to accelerate and enhance the induction of vaccine-specific responses [8]–[10], [21]. The major goal of the current study was to characterise the potential of the novel protein-particle HBSS1 vaccine in combination with a TLR-based [CpG or poly(I:C) alone] or mixed [alum and CpG or alum with poly(I:C)] adjuvant.

First, immunisation of HBSS1 with CpG and alum adjuvant combinations was evaluated in C57BL/6 mice. It is well known that alum induces a Th2-type immune response, interferes with cell-mediated immunity and blocks activation of CD8^+^CTL [7], [22]. CpG is a highly active TLR9 agonist that has been shown to act as a potent adjuvant, inducing Th-1 immunity. CpG ODNs are recognised by TLR9 molecules expressed by B cells and plasmacytoid dendritic cells (pDCs). CpG ODNs induce the Th1-acquired immune response and B-cell proliferation characterised primarily by the production of pro-inflammatory and Th1-biased cytokines (including IL-1, IL-6, TNF-α, IFN-γand IL-12) [13], [23]–[24]. Previous study suggested a possible mechanism of CpG immune-enhancing effects–induction by CpG ODN of the expression of co-stimulatory molecules on Ag-presenting cells, driving B-cell isotype switching in the appropriate cytokine milieu [25]. Several groups have reported a synergistic response upon use of CpG together with alum. An earlier study of the anti-S titres in mice immunised with HBsAg found that antibody titres in mice immunised with both CpG ODN plus alum were 35-fold those of mice immunised with alum alone, indicating a strong synergistic interaction between CpG ODN and alum. Alum induces a Th2 humoral response (mostly IgG1) and no CTL response. In contrast, CpG ODN induces a strong Th1 response with predominantly IgG2a Abs and CTLs, even when mixed with alum. Indeed, mice immunised with 20 µg CpG DNA and regular vaccine (containing alum adjuvant) had the highest concentration of antibody, IL-10 and IL-12 production, and activation of CD80 and CD86 molecules [26]. The Ab response was significantly improved in macaques and human clinical trials upon co-administration of CpG with Engerix B (the human hepatitis B vaccine), further confirming the adjuvant activity of CpG [27]–[32]. In this study, we obtained similar results when we immunised mice with HBSS1 and CpG ODN plus alum. Two weeks after the first immunisation, higher anti-S seroconversion was detected in the HBSS1 with CpG group and with mixed adjuvant groups than in the HBSS1 alone or HBSS1 with alum groups. Higher anti-PreS1 seroconversion was observed in all HBSS1 with adjuvant groups than that in the HBSS1 alone group. IgG subtype analysis after the second immunisation indicated that HBSS1 formulated with alum induced only Th2-type responses, whereas immunisation with CpG induced Th1-type responses. The mixed adjuvant formulation (CpG combined with alum) not only induced higher anti-S and -PreS1 titres, but also resulted in an IgG2/IgG1 ratio >1, indicating a Th1-biased immune response. ELISpot assay data showed a significantly enhanced antigen-specific cellular immune response when the frequencies of IFN-γ-producing cells were evaluated. The specific cellular immune responses (IFN-γELISpot analysis) were low (<50 SFC/10^6^ spleen cells) after two HBSS1 immunisations with adjuvant; however, after boosting with rAdSS1, the S and PreS1 antigen-specific SFC readings in all groups were significantly increased. Additionally, the SFC readings were higher in the HBSS1 with mixed adjuvants (A+C) group than those in the other groups, including the HBSS1 alone and with a single adjuvant [alum, CpG or Poly(I:C)] groups. The cytokine release assay detected no significantly greater cytokine production in the Ag-specific CD4^+^ and CD8^+^ T cells in the alum/CpG combined group. These results indicated that alum and CpG in combination both improved anti-S and -PreS1 titres and enhanced the number of Ag-specific T cells.

Second, we focused on the adjuvant activity of the poly(I:C)/alum combinations for HBSS1 priming. Poly(I:C) is a TLR-based immune adjuvant that binds TLR3 to activate the human innate immune system, with subsequent regulation of adaptive immunity [33]. Poly(I:C) has been used as an adjuvant for improving the humoral and cellular immunity induced by protein-based vaccines by means of inducing IL-12 and type I IFNs. Type I IFNs enhance DC maturation and B cell activation, and lead to the induction of potent CD4^+^ T cell and humoral immune responses, in mice immunised with protein antigens [34]. Finally, type I IFN is critical for cross-presentation of protein antigens to CD8^+^ T cells to generate cytotoxic T cells [15], [35]. Cross-presentation is also enhanced by the type I IFNs induced following TLR3 stimulation. Therefore, Poly(I:C), an agonist for TLR3, is considered an excellent adjuvant candidate because it induces a strong cellular immune response [36]. In our study, greater anti-S and -PreS1 seroconversion were induced in C57BL/6 mice immunised by HBSS1 with Poly(I:C) alone or with Poly(I:C)/alum mixed adjuvant, compared with that induced by HBSS1 alone or with alum. Additionally, the IgG (anti-S and anti-PreS1) subtype was primarily that of the IgG2 pattern, with (IgG2a+ IgG2b)/IgG1>1 in the HBSS1 with Poly(I:C) or Poly(I:C)/alum immunised groups. Furthermore, the cytokine release assay for Ag-specific CD4^+^ and CD8^+^ T cells confirmed that alum combined with poly(I:C) is highly immunogenic, and generated greater CD4^+^ and CD8^+^ T cell responses and Th1 cytokine (IFN-γ and IL-2) levels.

In conclusion, the novel HBSS1 protein vaccine in combination with CpG or poly(I:C) and alum adjuvant combinations represents a promising choice for HBV therapeutic vaccination. First, our data demonstrated the advantages of combination of two adjuvants, namely Poly(I:C) and alum, for induction of both T-cell and antibody responses. Alum/poly(I:C) combinations enhanced HBSS1-induced immunity, and generated a Th1-biased response (facilitated IgG isotype switching, resulting in more IFN-γ and IL-2 secretion), suggesting this immune regimen to be a good candidate for HBV therapeutic vaccination. Second, we demonstrated that priming with protein/adjuvant followed by a recombinant adenoviral-based vaccine boost maximises B and T-cell immunity. Taken together, our data support the use of combination alum and TLR ligands or agonists as a protein adjuvant for future HBV therapeutic vaccine trials, in combination with a viral vector boost. Our results may pave the way for therapeutic vaccination against HBV infection. Moreover, this strategy might be applicable to other persistent infections such as human immunodeficiency virus or tuberculosis.
